# Hyoscyamia

**Published:** 1880-09

**Authors:** Daniel R. Brower


### ARTICLE IV.

### HYOSCYAMIA. By DANIEL R. BROWER, M.D.

This alkaloid of Hyoscyamus, which can now readily be obtained in a state of purity, is a valuable addition, made recently, to the armamentarium of the physician, containing, as it does, in small and agreeable compass the hypnotic, anodyne and anti-spasmodic properties of the bulky and disagreeable preparations of hyoscyamus. The pure alkaloid occurs in needle-shaped crystals, and is odorless. The taste is bitter. It is quite insoluble in cold water; easily soluble in hot water and alcohol, ether and chloroform. It has, in my experience, great value in the treatment of mania, delirium tremens and paralysis agitans. In the treatment of mania it is particularly useful, inasmuch as the dose is small and its taste can readily be concealed with coffee, tea, or milk, and therefore is readily administered to those patients who positively refuse all ordinary medication.

I have given the drug recently in three cases of puerperal mania, with the effect of promptly modifying the mentality, to such an extent as to make possible the use of ordinary treatment. The preparation used was Merck's crystalized alkaloid, and the dose found effective much smaller than that recommended by recent writers; the one-thirtieth of a grain has been the ordinary dose employed. This dose has invariably produced mydriasis, and dryness of mouth and throat.

In the case of Mrs. B., suffering with puerperal mania, who was persistently refusing food and medicine, and at times quite violent in manner, the drug produced a marked change in mental condition. The violence gave place to calmness, and the desire for food returned. It did not, however, produce sleep, but advantage was taken of the pacification to administer by rectum a full dose (40 grains) of chloral hydrate, which was promptly followed by a long sleep and marked improvement in mentality. In about twenty-four hours after this there was evidence of the approach of another paroxysm of mania, which was arrested by the same method. The drug was then continued for several days, in doses just sufficient to produce a mild effect upon the pupil, the chloral injection given at bed time, and an abundance of milk and other nutritious food administered. The patient made a good recovery.

CASE II.—Mrs. N., a case of puerperal mania, complicated with metro-peritonitis. She had for twenty-four hours refused all food and medicine, and was found to be in a state of wild excitement. She was ordered hyoscyamia in one-thirtieth grain doses every four hours, and shortly after the third dose was administered she was found to be perfectly passive in mind and body, and took food without difficulty. She then received a forty-grain rectal injection of chloral, with forty minims of tinctura opii deodorata. This was followed by a profound sleep, from which she awoke with improvement in mental condition. The hyoscyamia was continued at longer intervals and tinctura opii deodorata used by rectal injection in sufficient doses to subdue the pain of the pelvic inflammation. The mania subsided, but death ensued in the fifth week after parturition.

CASE III.—Mrs. P., a mild case of puerperal mania without any pelvic complication, but like the other, refusing food and medicine. Hyoscyamia (1-30 grain) was administered in ice-water, and repeated in two hours in the same vehicle. In one hour after second dose muscular relaxation and pacification of mind were manifest, and very soon a profound sleep followed. The patient was continued under the influence of the drug for two weeks with a gradual subsidence of mental derangement and a restoration of general health.

My experience with the drug in delirium tremens is confined to a single case, in which there was marked cardiac irregularity and dyspnœa, so much so that I was afraid to administer chloral or the bromides. I gave this patient hyoscyamia in combination with digitalis in coffee, in the same dose above indicated, and closely watched the effect on the pulse. From a study of the physiological action of the mydriatic, I felt confidence in it as a respiratory and cardiac stimulant. The first dose had no effect upon the mental condition, but did improve the respiration and circulation. A second dose was administered in one hour, and was shortly followed by a subsidence of the excessive mental and muscular activity, sleep followed and a continuance of the treatment, giving just enough hyoscyamia to show its effects in a positive manner upon pupil, with liberal use of milk, beef tea and capsicum, resulted in restoration of patient, the result being in every way more favorable than opium, chloral or the bromides have ever produced in my experience.

In one case of paralysis agitans, the drug has served to diminish the tremor, and seemingly to arrest the progress of the disease. This patient is now taking about one-fortieth of a grain daily. This amount produced the characteristic pasty condition of the mouth, dilatation of the pupil and paralysis of accommodation. The one-twenty-fourth of a grain produced in this patient severe pains and spasmodic movements of the lower extremities, followed by complete muscular relaxation, and the effect on the eyes and the locomotion continued for forty-eight hours. The tremor is controlled by the drug very markedly, but there has been no permanent improvement. When he omits the drug for two or three days the disease shows itself with all its former intensity. The relief the remedy gives to the tremor without in any way disturbing the general system, except so far as the ocular phenomena are concerned, renders the drug exceedingly acceptable to him.

I have also found the drug of benefit in cholera, but, so far, am unable to say how far its influence is curative. It has been given with tonics, with stimulants, and with anti-rheumatics, but if the theory be correct that this disease is usually the result of congestive hyperæmia of the motor centers, we can see how it must produce good results.

As I said in the beginning, the great value of the drug consists in its furnishing, in a small compass, a powerful agent for overcoming the cerebral irritability and hyperæmia of mania, and is an agent that can, usually, be administered without the knowledge of the patient. Moreover, my experience with the drug, that is with Merck's crystalized alkaloid, would lead me to apprehend fatal results from the administration of one-half or one-quarter grain doses.

